# Supplementary material for: Cortical recruitment determines learning dynamics and strategy
Source: Nat Commun. 2019 Apr 1;10:1479. doi: 10.1038/s41467-019-09450-0 (PMC6443669; doi:10.1038/s41467-019-09450-0)
Supplement: Supplementary file 1 — Supplementary Information [file 41467_2019_9450_MOESM1_ESM.pdf]

Cortical recruitment determines learning dynamics and strategy

Ceballo et al.

Supplementary Figures

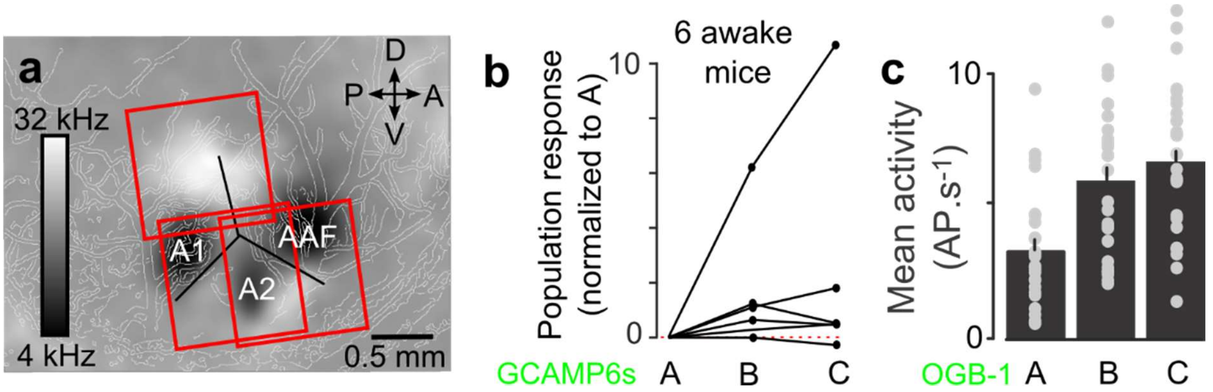

Supplementary Figure 1: Cortical recruitment differences are robust across mice and experiments

a. Localization and horizontal extent of the imaging fields-of-view (red squares) in one of the imaged mouse. The 1x1 mm fields-of-view were localized with blood vessels patterns (white lines). The auditory cortex is identified by subtracting intrinsic imaging responses to 4kHz (black colors) and 32 kHz (white colors) tones revealing the main tonotopic gradients. **b**. Responses of neural population recorded in each of the 6 awake mice included in the averages shown in presented in Figure 1 (activity is measured with GCAMP6s). Responses are measured as mean deconvolved calcium signals during the entire duration of the response (sound duration +0.5s). Normalization in obtained by subtracting and dividing with response to sound A. The distribution of responses to B and C are significantly above zero (n=6 mice, Wilcoxon signed rank test, p=0.031 for B and C) **c**. Average population activity in OGB1 labelled neurons imaged with two-photon microscopy in 7 isoflurane anesthetized mice (28 population, 1994 neurons) for sounds A, B and C. Mean ± SEM, for A: 3.1176 ± 0.3726 AP.s^{-1}, B: 5.4527 0.4982 AP.s^{-1}, C: 6.3453 ± 0.4978 AP.s^{-1}. Responses to sound B and C are significantly different from A, but not from each other Wilcoxon rank sum test, p=0.0012, p=1.5×10^{-5}, p=0.31. Error bars represent standard errors (SEM).

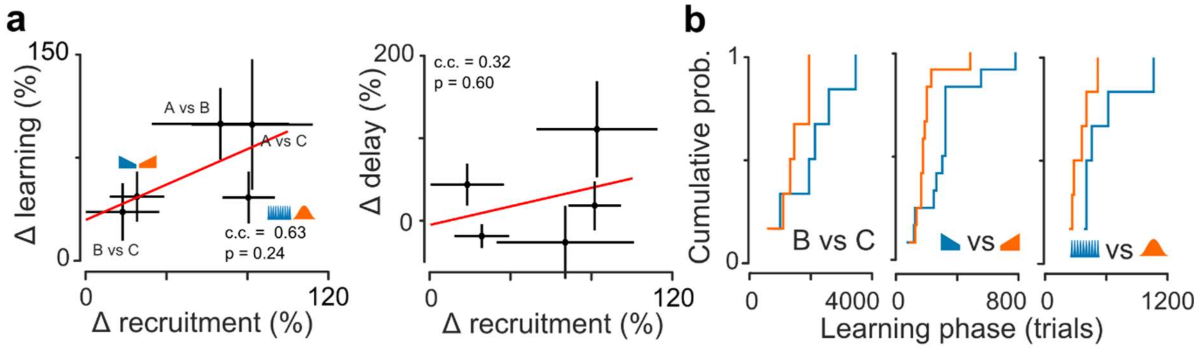

Supplementary Figure 2: Impact of cortical recruitment on learning and delay phase durations.

a. Mean +/- standard error of the difference of learning (left) and delay (right) phase durations plotted against the difference in neural recruitment by the stimuli. Correlation coefficients: 0.63 (p=0.24, n=5) for the learning phase, 0.32 for the delay phase (p=0.60, n=5). Note that high correlations are expected to occur by chance for low sample size. **b.** Cumulative distributions of learning phase durations for sound pairs B-C, for the up- (orange symbol) and down-ramp (blue symbol) and for the 1 Hz (orange symbol) and 20Hz (blue symbol) modulated sounds.

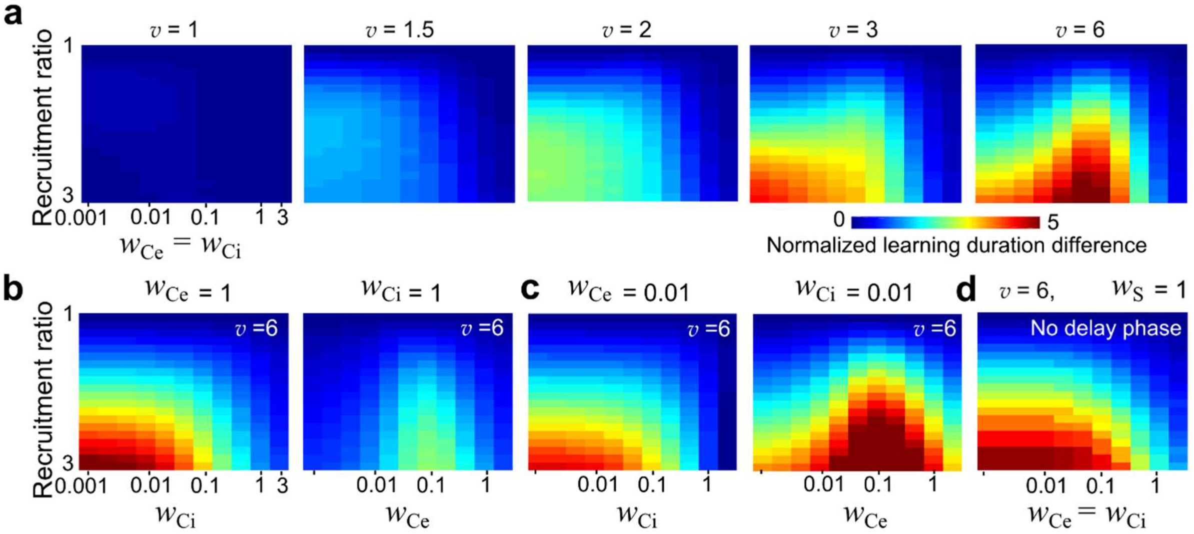

Supplementary Figure 3: The impact of neuronal recruitment on learning speed depends on the initial strengths of synaptic connections in the model

a. Normalized learning phase duration difference (duration S- with more recruitment - duration S- with more recruitment)/(duration S- with more recruitment) is color-coded and plotted against the

cortical recruitment ratio of the discriminated stimuli and the strength of the initial inhibitory and excitatory connection to unspecific cues (w_{Ci} and w_{Ce}, same value for both). From left to right the value of the asymmetric learning rate parameter v is increased. Simulation parameters: $w_S = 0.01$, $\alpha = 0.01$, $\sigma = 0.6195$. **b.** Same as **a**, but varying only the initial inhibitory (left) or excitatory connection (right) while the other connection is kept constant at value 1. **c.** Same as **b**, but with the non-varied initial connection kept at a constant value of 0.01. **d.** Same as **a**, but now with initial connectivity from sound stimuli $w_S = 1$ such that there is no delay phase. In all plots, the recruitment ratio is equal to the larger of the two saliency values.

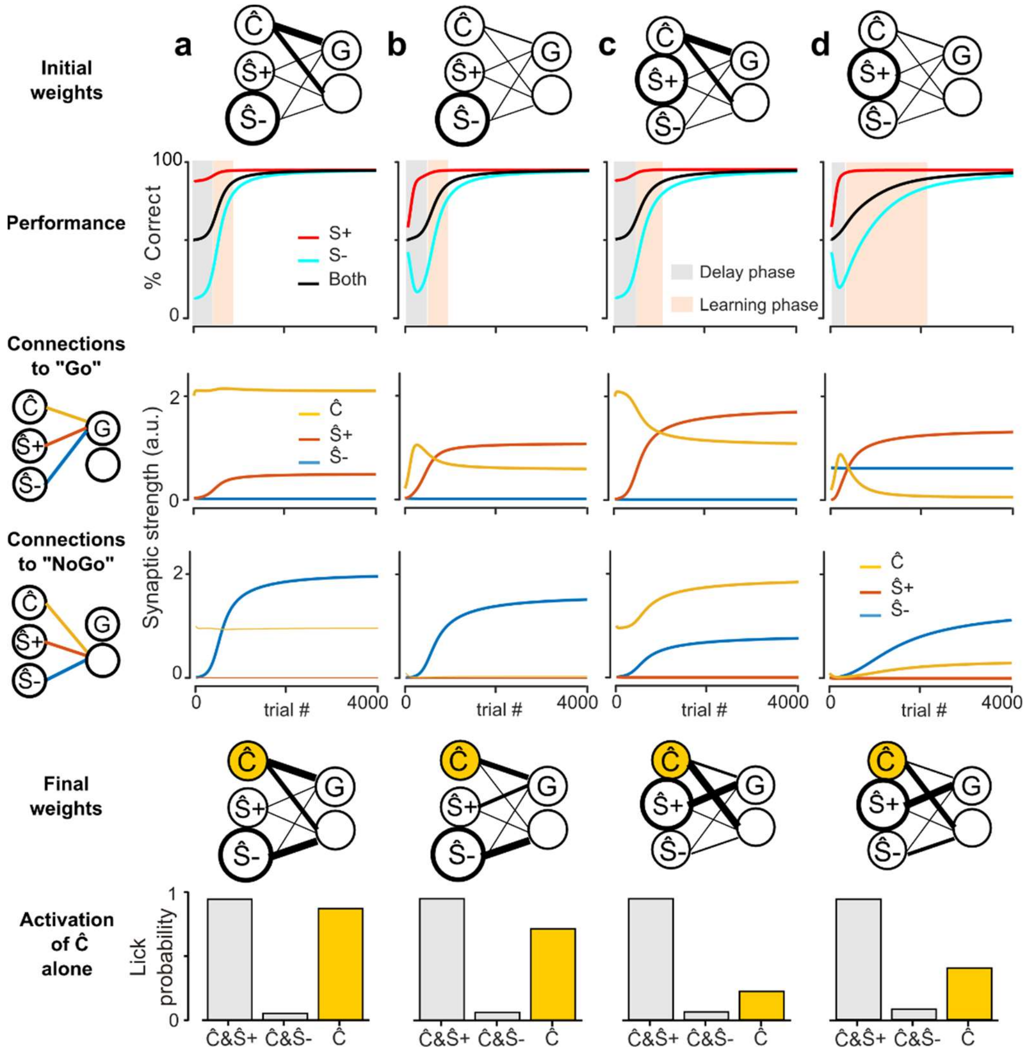

Supplementary Figure 4: The effects of neuronal recruitment in the model do not crucially depend on the magnitude of recruitment differences.

Same simulations as in **Fig. 5**, but with a ratio of recruitment between the least and the most active stimulus equal to 1.4 (corresponding to the ratio of numbers of recruited neurons by sounds C and A) instead of 2 (chosen according to the ratio of population firing rates for sounds C and A). Simulation parameters: $\mathbf{X} = [1 ; 1; 1.4]$, $\alpha = 0.01$, $\sigma = 0.6195$, $v = 6$, $w_{Ci} = 1$, $w_{Ce} = 2$, and $w_S = 0.01$. **b.** Same as **a**, but with $w_{Ci} = 0.1$, $w_{Ce} = 0.2$. **c.** Same as **a**, but with $\mathbf{X} = [1 ; 1.4 ; 1]$. **d.** Same as **c**, but with $w_{Ci} = 0.1$, $w_{Ce} = 0.2$.

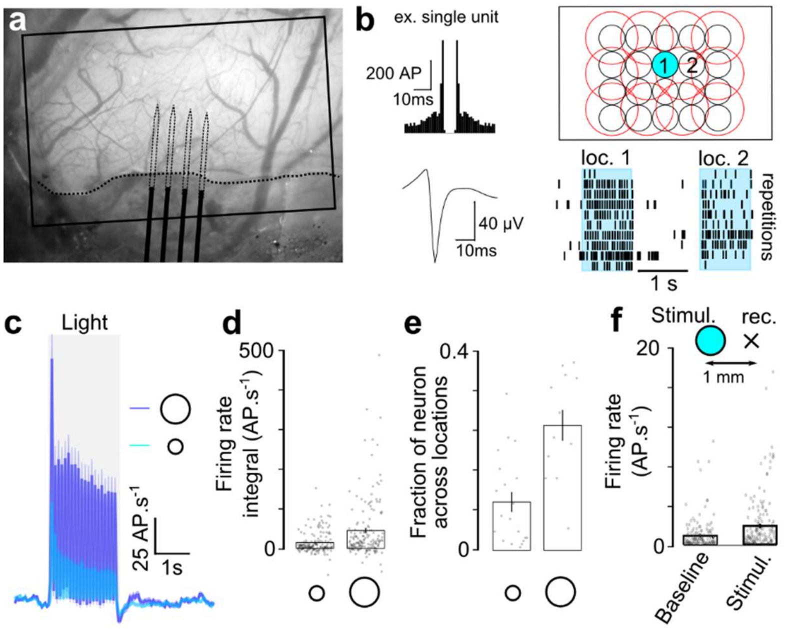

Supplementary Figure 5: Cortical responses to patterned optogenetic stimulations.

a. Picture of the electrophysiolocal experiment setting. A 4-shanks silicon probe (sketched) is inserted with a shallow angle through a hole under the glass window (curved dashed line). The rectangle indicates the area covered by the videoprojector. **b.** Example waveform (bottom left) and spike train autocorrelogram (top left) of a single unit. An array of 3 x 4 stimulus locations (spacing 480 µm) was probed to reconstruct the spread of activity for the large disk (red, top right), and 4 x 5 locations (out of 5 x 8) were selected for the small disk in order to cover a similar extent as the grid of large disk centers. Example raster plots for two locations are shown at the bottom right. **c.** Mean response time course for all selected locations and single units (160 single units, 2 mice, 5 recording locations), for the small (light blue) and large (dark blue) disk. **d**. Integral of population (bars) and single units (gray dots) firing rate computed by summing over the grids of stimulus location for the small and large disk, in order to estimate the total response elicited by one disk across auditory cortex. The small disk elicits about 3 times less activity than the large disk (small 13.7 ± 2.0 $AP.s^{-1}$; large: 42.3 ± 5.7 $AP.s^{-1}$, n = 160 cells, in 2 mice). Thus, the recruitment difference (as measured for sounds in **Fig. 4b** and **Supplementary Fig. 2a**) between the strong (1 small + 1 large disk) and weaker (2 small disks) stimuli is equal to $2*(S_{strong}-S_{weak})/(S_{strong}+S_{weak})$ = 69%. **e**. Average fraction of responsive neurons over the 160 neurons recorded across the same range of grid location centers for the small and large disks.

Grey dots indicate the fraction of responsive neurons for individual locations. The small disk activated about 2.5 times less neurons than the large disk (small 10% ± 2%, n = 20 locations; large: 25 ± 3 %, n = 12 locations). Thus, the recruitment difference between the stronger and weaker stimuli is equal to 55%. **f.** Average activity recorded at the relative position of the Ĉ spot (1 mm away, Fig. 6) with respect to the center of the large disk (estimated as the stimulus location that elicits the best response) before large disk stimulation (baseline) and during stimulation (stimul.). A residual positive response is observed at the Ĉ spot location (baseline 0.87 ± 0.12 $AP.s^{-1}$, stimul. 1.89± 0.22 $AP.s^{-1}$, n = 160 cells). Error bars represent standard errors (SEM).

Supplementary note 1

Introduction

The purpose of this note is to provide mathematical demonstrations of some of the results obtained in simulations in order to show their generality. The analytical arguments are presented in three parts. (i) Starting from the standard equations, we introduce a change of variables which permits to observe the model dynamics in a convenient coordinate system. (ii) We use this coordinate system to elaborate a geometrical interpretation of the learning process. Based on this interpretation, we demonstrate that learning increases synaptic weights in order to compensate for the difference of neural recruitment in the input stimulus. (iii) Finally, we show the generality of the neuronal recruitment dependence of the response to the selective activation of the common unit, as exemplified in simulation in Figure 5 and as experimentally observed in Figure 6.

1 Model overview

As detailed in the main text, the model consists of three neural populations whose activity is described in a vector $\mathbf{X}$. We note $\mathbf{X}_1 = \begin{bmatrix} \hat{C} \\ \hat{S}^+ \\ 0 \end{bmatrix}$ the vector that describes the input corresponding to the rewarded stimulus and $\mathbf{X}_2 = \begin{bmatrix} \hat{C} \\ 0 \\ \hat{S}^- \end{bmatrix}$ the vector corresponding to the non-rewarded stimulus.
The model describes the probability for the mouse to lick (y= 1) after the rewarded stimulus as:

$$p(y=1|\mathbf{X}=\mathbf{X}_1) = < y_1 > = \frac{1}{2} + \frac{1}{2}\,\mathrm{erf}\left(\frac{<\Delta\mathbf{W}>^t .\mathbf{X}_1}{\sqrt{2}}\right) \tag{1}$$

and the probability to withhold licking after the non-rewarded stimulus as:

$$p(y=0|\mathbf{X}=\mathbf{X}_2) = 1 - p(y=1|\mathbf{X}=\mathbf{X}_2) = 1 - < y_2 > = \frac{1}{2} - \frac{1}{2}\,\mathrm{erf}\left(\frac{<\Delta\mathbf{W}>^t .\mathbf{X}_2}{\sqrt{2}}\right) \tag{2}$$

The critical variable for the behavioral output is the vector $\Delta\mathbf{W} = \mathbf{W}_E - \mathbf{W}_I$, which evolves according to the following system of equations:

$$\begin{cases} \delta < \mathbf{W}_E > = \frac{\alpha}{2} < \mathbf{W}_E > \odot \Big(\mathbf{X}_1.f(1-\sigma<\Delta\mathbf{W}>^t.\mathbf{X}_1) < y_1 > + \mathbf{X}_2.f(-1-\sigma<\Delta\mathbf{W}>^t.\mathbf{X}_2) < y_2 > \Big) \\ \delta < \mathbf{W}_I > = -\frac{\alpha}{2} < \mathbf{W}_I > \odot \Big(\mathbf{X}_1.f(1-\sigma<\Delta\mathbf{W}>^t.\mathbf{X}_1) < y_1 > + \mathbf{X}_2.f(-1-\sigma<\Delta\mathbf{W}>^t.\mathbf{X}_2) < y_2 > \Big) \end{cases} \tag{3}$$

in which $\odot$ is a Hadamard (point-wise) vector product. The function f is a piece-wise linear function: $f(x) = \nu x, \nu > 1, x > 0$ and $f(x) = x$ for $x < 0$.

To describe the dynamic of $\Delta\mathbf{W}$, we simply re-write the precedent system in the basis $\{\Delta\mathbf{W}, \Sigma\mathbf{W}\} = \{\mathbf{W}_E - \mathbf{W}_I, \mathbf{W}_E + \mathbf{W}_I\}$ obtaining:

$$\begin{cases} \delta < \Delta\mathbf{W} > = \frac{\alpha}{2} < \Sigma\mathbf{W} > \odot \Big(\mathbf{X}_1.f(1-\sigma<\Delta\mathbf{W}>^t.\mathbf{X}_1) < y_1 > + \mathbf{X}_2.f(-1-\sigma<\Delta\mathbf{W}>^t.\mathbf{X}_2) < y_2 > \Big) \\ \delta < \Sigma\mathbf{W} > = \frac{\alpha}{2} < \Delta\mathbf{W} > \odot \Big(\mathbf{X}_1.f(1-\sigma<\Delta\mathbf{W}>^t.\mathbf{X}_1) < y_1 > + \mathbf{X}_2.f(-1-\sigma<\Delta\mathbf{W}>^t.\mathbf{X}_2) < y_2 > \Big) \end{cases} \tag{4}$$

Interestingly, although the Hadamard product does not create interactions between the dimensions of the weight vector, the temporal evolutions of the dimensions are interdependant through the dot product involving $\Delta\mathbf{W}$. The reward R minus the expectations $\sigma < \Delta\mathbf{W} >^t .\mathbf{X}_{1,2}$ gives the prediction errors for the rewarded $\mathbf{X}_1$ and non rewarded stimuli $\mathbf{X}_2$. Non-zero prediction errors drive learning. The model stops learning onces both prediction error terms are nul, leading to the equation that the steady state synaptic weight solution $< \Delta\mathbf{W} >$ has to fulfill:

$$\begin{cases} \sigma^{-1} = < \Delta\mathbf{W} >^t .\mathbf{X}_1 \\ \sigma^{-1} = < \Delta\mathbf{W} >^t .(-\mathbf{X}_2) \end{cases} \tag{5}$$

Importantly, this equation has not a unique solution, because the only components of $< \Delta\mathbf{W} >$ that are relevant in equation (5) are those that are not orthogonal to the vectors $\mathbf{X}_1$ and $\mathbf{X}_2$.

In this study, $\mathbf{X}_1$ and $\mathbf{X}_2$ are non-colinear and thus define a two-dimensional plane in the three dimensional space in which they live. We note $(\Delta\mathbf{W})^{II}$ the paralell component of $< \Delta\mathbf{W} >$ contained in the plane $< \mathbf{X}_1, \mathbf{X}_2 >$ and we note

$(\Delta\mathbf{W})^{\perp}$ its orthogonal complement. The connectivity that maximizes the performance lives in a **line attractor** that can be written as:

$$<\Delta\mathbf{W}>=(\Delta\mathbf{W})^{II}+a(\Delta\mathbf{W})^{\perp}, a\ real$$

To obtain the solution of equation (5), we decompose the parallel component along the input vectors $(\Delta\mathbf{W})^{II}=\alpha_1\frac{\mathbf{X}_1}{||\mathbf{X}_1||}-\alpha_2\frac{\mathbf{X}_2}{||\mathbf{X}_2||}$ and replace in equation (5):

$$\begin{cases}\sigma^{-1}=\alpha_1.\dfrac{\mathbf{X}_1^2}{||\mathbf{X}_1||}-\alpha_2\dfrac{\mathbf{X}_1.\mathbf{X}_2}{||\mathbf{X}_2||}\\ \sigma^{-1}=\alpha_2.\dfrac{\mathbf{X}_2^2}{||\mathbf{X}_2||}-\alpha_1\dfrac{\mathbf{X}_1.\mathbf{X}_2}{||\mathbf{X}_1||}\end{cases}\tag{6}$$

Solving the linear system, we can express the solutions as a function of relative angle ψ and the norms of $\mathbf{X}_1$ and $\mathbf{X}_2$:

$$\begin{cases}\alpha_1=\dfrac{\sigma^{-1}}{||\mathbf{X}_1||\sin^2\psi}\left(1+\dfrac{||\mathbf{X}_1||}{||\mathbf{X}_2||}\cos\psi\right)\\ \alpha_2=\dfrac{\sigma^{-1}}{||\mathbf{X}_2||\sin^2\psi}\left(1+\dfrac{||\mathbf{X}_2||}{||\mathbf{X}_1||}\cos\psi\right)\end{cases}\tag{7}$$

Steady state performance can be obtained based on the steady state connectivity, by plugging equations (5) into (1) and (2) to get:

$$\begin{cases}p(y=1|\mathbf{X}=\mathbf{X}_1)=\dfrac{1}{2}+\dfrac{1}{2}erf(\dfrac{\sigma^{-1}}{\sqrt{2}})\\ p(y=0|\mathbf{X}=\mathbf{X}_2)=\dfrac{1}{2}+\dfrac{1}{2}erf(\dfrac{\sigma^{-1}}{\sqrt{2}})\end{cases}\tag{8}$$

Interestingly, equation (8) shows that the same asymptotic performance is reached for the S^+ and for S^- stimulus, which is easy to verify in simulations.

2 Learnt synaptic weights compensate for neuronal recruitment differences

We can now use the equations derived above to study the effect of asymmetric neuronal recruitment on learning.

An intuitive way of understanding the learning process is to consider the 2D plane formed by $\mathbf{X}_2$ and $\mathbf{X}_1$. During learning, the parallel component of the synaptic weight vector $\Delta\mathbf{W}$ changes to reach its steady state position. When the S^+ and S^- stimuli trigger equal neuronal recruitment i.e. when $||\mathbf{X}_1||=||\mathbf{X}_2||$ equation (5) tells us that the projection of $\Delta\mathbf{W}$ is identical onto both $\mathbf{X}_1$ and $-\mathbf{X}_2$. Therefore $(\Delta\mathbf{W})^{II}$ is the bisector of the angle formed by vectors $\mathbf{X}_1$ and $-\mathbf{X}_2$. This can also be seen in equation (7): if $||\mathbf{X}_1||=||\mathbf{X}_2||$, then $\alpha_1=-\alpha_2$.

If $||\mathbf{X}_1||\neq||\mathbf{X}_2||$ and $\psi\notin\{0,\pi\}$, one can intuitively understand that to have equal projections on $\mathbf{X}_2$ and $\mathbf{X}_1$ (i.e. fulfill equation 5), the parallel component of the steady state weight vector has to rotate closer to the shortest of the two input vectors. This can also be easily demonstrated using equation (7) to express the ratio of the projections α_1 and α_2 of $\Delta\mathbf{W}$ on the input vectors $\mathbf{X}_2$ and $\mathbf{X}_1$ (i.e. the effective synaptic weights between the stimuli and the decision unit):

$$\frac{\alpha_1}{\alpha_2}=\frac{||\mathbf{X}_2||}{||\mathbf{X}_1||}\frac{1+\frac{||\mathbf{X}_1||}{||\mathbf{X}_2||}\cos\psi}{1+\frac{||\mathbf{X}_2||}{||\mathbf{X}_1||}\cos\psi}=\frac{\frac{||\mathbf{X}_2||}{||\mathbf{X}_1||}+\cos\psi}{1+\frac{||\mathbf{X}_2||}{||\mathbf{X}_1||}\cos\psi}\tag{9}$$

It is easy to show (by computing the derivative) that $\frac{\alpha_1}{\alpha_2}$ is monotonically growing with $\frac{||\mathbf{X}_2||}{||\mathbf{X}_1||}$ And it is clear that $\frac{\alpha_1}{\alpha_2}=1$ when $\frac{||\mathbf{X}_2||}{||\mathbf{X}_1||}=1$. Therefore:

$$\begin{cases}||\mathbf{X}_2||<||\mathbf{X}_1||\iff\alpha_2>\alpha_1\\ ||\mathbf{X}_2||>||\mathbf{X}_1||\iff\alpha_2<\alpha_1\end{cases}\tag{10}$$

Thus, we have shown in a general manner that when one stimulus recruits less activity than the other, the synaptic weight associated to the weaker stimulus is increased through learning in order to compensate for the neuronal recruitment discrepancy.

3 Model prediction: response to activation of the 'common' input population alone depends on activity recruited in the specific input populations

The compensation of neuronal recruitment differences by learnt synaptic weights has a very interesting consequence for the $\hat{C}$ neural population that encodes information common to the S^+ and S^- stimuli. If we recall that $(\Delta\mathbf{W})^{II}=\alpha_1\frac{\mathbf{X}_1}{||\mathbf{X}_1||}-\alpha_2\frac{\mathbf{X}_2}{||\mathbf{X}_2||}$, then we can express the learnt synaptic weight for the dimension correxponding to $\hat{C}$:

$$(\Delta \mathbf{W})^{II}_{\hat{C}} = \hat{C} \left(\frac{\alpha_1}{||\mathbf{X}_1||} - \frac{\alpha_2}{||\mathbf{X}_2||} \right) \tag{11}$$

Based on (10), if $||\mathbf{X}_2|| > ||\mathbf{X}_1||$ then $\alpha_1 > \alpha_2$ and thus $\frac{\alpha_1}{||\mathbf{X}_1||} > \frac{\alpha_2}{||\mathbf{X}_2||}$. Similarly, if $||\mathbf{X}_2|| < ||\mathbf{X}_1||$ then $\frac{\alpha_1}{||\mathbf{X}_1||} < \frac{\alpha_2}{||\mathbf{X}_2||}$. Hence the sign of the learnt synaptic weights $(\Delta \mathbf{W})^{II}_{\hat{C}}$ between the common neural population and the decision unit is equal to the sign of the neuronal recruitment difference $||\mathbf{X}_2|| - ||\mathbf{X}_1||$. Thanks to equation (1), this can be translated into response probabilities when the common population is activated alone to probe the steady state solution:

$$\begin{cases} ||\mathbf{X}_2|| > ||\mathbf{X}_1|| \Rightarrow p(y = 1|\mathbf{X} = \mathbf{X}_C) > \dfrac{1}{2} \\ ||\mathbf{X}_2|| < ||\mathbf{X}_1|| \Rightarrow p(y = 1|\mathbf{X} = \mathbf{X}_C) < \dfrac{1}{2} \end{cases} \tag{12}$$

This demonstrates that, whatever the choice of parameters in the model, if the S^- stimulus recruits more activity than the S^+ stimulus, probing the common population alone will lead to a biais towards the positive response. On the contrary, if the S^+ stimulus is recruits more actvity than the S^- stimulus, probing the common population alone will lead to a bias towards the negative response. This generic property corresponds exactly to the effect observed in our simulation (main text: Figure 5) and in mice after learning to discriminate two overlapping artificial cortical patterns recruiting different amounts of cortical activity (main text: Figure 6).
