# Supplementary material for: Cortical recruitment determines learning dynamics and strategy
Source: Nat Commun. 2019 Apr 1;10:1479. doi: 10.1038/s41467-019-09450-0 (PMC6443669; doi:10.1038/s41467-019-09450-0)
Supplement: Supplementary file 3 — Reporting Summary [file 41467_2019_9450_MOESM3_ESM.pdf]

nature research

Corresponding author(s): *Double-blind peer review submissions: write DBPR and your manuscript number here instead of author names.*

Last updated by author(s): *YYYY-MM-DD*

# Reporting Summary

Nature Research wishes to improve the reproducibility of the work that we publish. This form provides structure for consistency and transparency in reporting. For further information on Nature Research policies, see Authors & Referees and the Editorial Policy Checklist.

## Statistics

For all statistical analyses, confirm that the following items are present in the figure legend, table legend, main text, or Methods section.

| n/a | Confirmed | |
|---|---|---|
| ☐ | ☒ | The exact sample size (*n*) for each experimental group/condition, given as a discrete number and unit of measurement |
| ☐ | ☒ | A statement on whether measurements were taken from distinct samples or whether the same sample was measured repeatedly |
| ☐ | ☒ | The statistical test(s) used AND whether they are one- or two-sided<br>*Only common tests should be described solely by name; describe more complex techniques in the Methods section.* |
| ☐ | ☒ | A description of all covariates tested |
| ☐ | ☒ | A description of any assumptions or corrections, such as tests of normality and adjustment for multiple comparisons |
| ☐ | ☒ | A full description of the statistical parameters including central tendency (e.g. means) or other basic estimates (e.g. regression coefficient) AND variation (e.g. standard deviation) or associated estimates of uncertainty (e.g. confidence intervals) |
| ☒ | ☐ | For null hypothesis testing, the test statistic (e.g. *F*, *t*, *r*) with confidence intervals, effect sizes, degrees of freedom and *P* value noted<br>*Give P values as exact values whenever suitable.* |
| ☒ | ☐ | For Bayesian analysis, information on the choice of priors and Markov chain Monte Carlo settings |
| ☒ | ☐ | For hierarchical and complex designs, identification of the appropriate level for tests and full reporting of outcomes |
| ☒ | ☐ | Estimates of effect sizes (e.g. Cohen's *d*, Pearson's *r*), indicating how they were calculated |

*Our web collection on statistics for biologists contains articles on many of the points above.*

## Software and code

Policy information about availability of computer code

| | |
|---|---|
| Data collection | Data collection is performed with commercial acquisition softwares from Femtonics and Intan |
| Data analysis | Data analysis is performed with custom software written in Matlab or Python that are freely available online available on the Dryad Digital Repository (http://datadryad.org/) doi:10.5061/dryad.47h8t87 or at https://www.bathellier-lab.org/downloads or are published (Imaging data preprocessing, https://doi.org/10.7554/eLife.26337.001) |

For manuscripts utilizing custom algorithms or software that are central to the research but not yet described in published literature, software must be made available to editors/reviewers. We strongly encourage code deposition in a community repository (e.g. GitHub). See the Nature Research guidelines for submitting code & software for further information.

## Data

Policy information about availability of data

All manuscripts must include a data availability statement. This statement should provide the following information, where applicable:

- Accession codes, unique identifiers, or web links for publicly available datasets
- A list of figures that have associated raw data
- A description of any restrictions on data availability

All datasets, analysis software and codes for running the simulations of our model are freely available on the Dryad Digital Repository (http://datadryad.org/) doi:10.5061/dryad.47h8t87 or  on https://www.bathellier-lab.org/downloads

# Field-specific reporting

Please select the one below that is the best fit for your research. If you are not sure, read the appropriate sections before making your selection.

☒ Life sciences ☐ Behavioural & social sciences ☐ Ecological, evolutionary & environmental sciences

For a reference copy of the document with all sections, see nature.com/documents/nr-reporting-summary-flat.pdf

# Life sciences study design

All studies must disclose on these points even when the disclosure is negative.

| | |
|---|---|
| Sample size | Sample size were chosen to be just above the minimum value required for non parametric test to be effective in detecting effects. Typically, n>=5. |
| Data exclusions | No data was excluded from the analysis. |
| Replication | At least 5 biological replicates of the main findings have been performed. |
| Randomization | No randomisation was performed as all data acquisition and behavioral procedures are performed via computer driven device without human intervention |
| Blinding | No blinding was used as all data acquisition and behavioral procedures are performed via computer driven device without human intervention |

# Reporting for specific materials, systems and methods

We require information from authors about some types of materials, experimental systems and methods used in many studies. Here, indicate whether each material, system or method listed is relevant to your study. If you are not sure if a list item applies to your research, read the appropriate section before selecting a response.

## Materials & experimental systems

| n/a | Involved in the study | |
|---|---|---|
| ☒ | ☐ | Antibodies |
| ☒ | ☐ | Eukaryotic cell lines |
| ☒ | ☐ | Palaeontology |
| ☐ | ☒ | Animals and other organisms |
| ☒ | ☐ | Human research participants |
| ☒ | ☐ | Clinical data |

## Methods

| n/a | Involved in the study | |
|---|---|---|
| ☒ | ☐ | ChIP-seq |
| ☒ | ☐ | Flow cytometry |
| ☒ | ☐ | MRI-based neuroimaging |

## Animals and other organisms

Policy information about studies involving animals; ARRIVE guidelines recommended for reporting animal research

| | |
|---|---|
| Laboratory animals | All mice used for imaging and behavior were 8 to 16 weeks old C57Bl6J and GAD2-Cre (Jax #010802) x RCL-TdT (Jax #007909) mice. Mice used for optogenetics were 8 to 16 weeks old males and female obtained by crossing homozygous Emx1IRES-cre (Jax #005628) mice with Ai27 (Jax # 012567) mice to obtain expression of Td-Tomato-tagged channelrhodopsin (ChR2) in excitatory neurons of the cortex. |
| Wild animals | N/A |
| Field-collected samples | N/A |
| Ethics oversight | All procedures were approved by the Austrian laboratory animal law guidelines (Approval #: M58 / 02182 / 2007 /11; M58 / 02063 / 2008 /8) and the French Ethical Committee (authorization 00275.01). |

Note that full information on the approval of the study protocol must also be provided in the manuscript.
